# Supplementary material for: Obesity phenotypes and dyslipidemia in adults from four African countries: An H3Africa AWI-Gen study
Source: PLoS One. 2025 Jan 30;20(1):e0316527. doi: 10.1371/journal.pone.0316527 (PMC11781721; doi:10.1371/journal.pone.0316527)
Supplement: S1 Checklist — (DOC) [file pone.0316527.s001.doc]

STROBE Statement—checklist of items that should be included in reports of observational studies

|  | Item No | Recommendation |
| --- | --- | --- |
| **Title and abstract** | 1 | (*a*) Indicate the study’s design with a commonly used term in the title or the abstract  **Response:** The study design was a cross-sectional study and this has been stated in line 79 on page 3. |
|  |  | (*b*) Provide in the abstract an informative and balanced summary of what was done and what was found  **Response:** The procedure and findings of the study are found in lines 79 to 92 of the abstract on page 3. |
| Introduction | | |
| Background/rationale | 2 | Explain the scientific background and rationale for the investigation being reported  **Response:** The scientific background and rational for the study are contained in lines 116 to 151 on pages 4 and 5. |
| Objectives | 3 | State specific objectives, including any prespecified hypotheses  **Response:** The objective of the study is stated in lines 151 to 154 on page 5. |
| Methods | | |
| Study design | 4 | Present key elements of study design early in the paper  **Response:** The study design is stated in lines 169 to 171 in the method section on page 6. |
| Setting | 5 | Describe the setting, locations, and relevant dates, including periods of recruitment, exposure, follow-up, and data collection  **Response:** These are contained in lines 169 to 193 on page 6. |
| Participants | 6 | (*a*) *Cohort study*—Give the eligibility criteria, and the sources and methods of selection of participants. Describe methods of follow-up  *Case-control study*—Give the eligibility criteria, and the sources and methods of case ascertainment and control selection. Give the rationale for the choice of cases and controls  *Cross-sectional study*—Give the eligibility criteria, and the sources and methods of selection of participants  **Response:** The eligibility criteria for the cross-sectional study are stated in lines 174 to 176 on page 6. |
|  |  | (*b*) *Cohort study*—For matched studies, give matching criteria and number of exposed and unexposed  *Case-control study*—For matched studies, give matching criteria and the number of controls per case |
| Variables | 7 | Clearly define all outcomes, exposures, predictors, potential confounders, and effect modifiers. Give diagnostic criteria, if applicable  **Response:** All variables and definitions are stated in lines 197 to 236 on pages 6 to 8. |
| Data sources/ measurement | 8* | For each variable of interest, give sources of data and details of methods of assessment (measurement). Describe comparability of assessment methods if there is more than one group  **Response:** The source of data and details of methods of assessment are contained in lines 192 to 236 on pages 6 to 8. |
| Bias | 9 | Describe any efforts to address potential sources of bias |
| Study size | 10 | Explain how the study size was arrived at  **Response:** The justification for the sample size is stated in lines 184 to 189 on page 6 |
| Quantitative variables | 11 | Explain how quantitative variables were handled in the analyses. If applicable, describe which groupings were chosen and why |
| Statistical methods | 12 | (*a*) Describe all statistical methods, including those used to control for confounding  **Response:** The description of the statistical methods is contained in lines 239 to 256 on pages 8 and 9. |
|  |  | (*b*) Describe any methods used to examine subgroups and interactions  **Response:** These are contained in lines 248 to 256 on pages 8 and 9. |
|  |  | (*c*) Explain how missing data were addressed |
|  |  | (*d*) *Cohort study*—If applicable, explain how loss to follow-up was addressed  *Case-control study*—If applicable, explain how matching of cases and controls was addressed  *Cross-sectional study*—If applicable, describe analytical methods taking account of sampling strategy |
|  |  | (*e*) Describe any sensitivity analyses |

Continued on next page

| Results | | |
| --- | --- | --- |
| Participants | 13* | (a) Report numbers of individuals at each stage of study—eg numbers potentially eligible, examined for eligibility, confirmed eligible, included in the study, completing follow-up, and analysed |
|  |  | (b) Give reasons for non-participation at each stage |
|  |  | (c) Consider use of a flow diagram |
| Descriptive data | 14* | (a) Give characteristics of study participants (eg demographic, clinical, social) and information on exposures and potential confounders  **Response**: The characteristics of the participants are described in lines 271 to 278 on page 9. |
|  |  | (b) Indicate number of participants with missing data for each variable of interest |
|  |  | (c) *Cohort study*—Summarise follow-up time (eg, average and total amount) |
| Outcome data | 15* | *Cohort study*—Report numbers of outcome events or summary measures over time |
|  |  | *Case-control study—*Report numbers in each exposure category, or summary measures of exposure |
|  |  | *Cross-sectional study—*Report numbers of outcome events or summary measures  ***Response:*** These are contained in ***l***ines 285 to 305 on pages 12 and 13. |
| Main results | 16 | (*a*) Give unadjusted estimates and, if applicable, confounder-adjusted estimates and their precision (eg, 95% confidence interval). Make clear which confounders were adjusted for and why they were included  **Response:** These are contained in lines 313 to 332 on pages 15 and 16. |
|  |  | (*b*) Report category boundaries when continuous variables were categorized  **Response:** These are contained in lines 312 to 324 on page 15. |
|  |  | (*c*) If relevant, consider translating estimates of relative risk into absolute risk for a meaningful time period |
| Other analyses | 17 | Report other analyses done—eg analyses of subgroups and interactions, and sensitivity analyses |
| Discussion | | |
| Key results | 18 | Summarise key results with reference to study objectives  **Response:** The summary of key findings are stated in lines 353 to 360 on pages 17 and 18. |
| Limitations | 19 | Discuss limitations of the study, taking into account sources of potential bias or imprecision. Discuss both direction and magnitude of any potential bias  **Response:** The limitations are stated in lines 428 and 429 on page 20. |
| Interpretation | 20 | Give a cautious overall interpretation of results considering objectives, limitations, multiplicity of analyses, results from similar studies, and other relevant evidence |
| Generalisability | 21 | Discuss the generalisability (external validity) of the study results  Response: |
| Other information | | |
| Funding | 22 | Give the source of funding and the role of the funders for the present study and, if applicable, for the original study on which the present article is based  **Response:** Information on funding source is contained in lines 445 to 454 on pages 20 and 21. |

*Give information separately for cases and controls in case-control studies and, if applicable, for exposed and unexposed groups in cohort and cross-sectional studies.

**Note:** An Explanation and Elaboration article discusses each checklist item and gives methodological background and published examples of transparent reporting. The STROBE checklist is best used in conjunction with this article (freely available on the Web sites of PLoS Medicine at http://www.plosmedicine.org/, Annals of Internal Medicine at http://www.annals.org/, and Epidemiology at http://www.epidem.com/). Information on the STROBE Initiative is available at www.strobe-statement.org.
