# Supplementary material for: Obesity phenotypes and dyslipidemia in adults from four African countries: An H3Africa AWI-Gen study
Source: PLoS One. 2025 Jan 30;20(1):e0316527. doi: 10.1371/journal.pone.0316527 (PMC11781721; doi:10.1371/journal.pone.0316527)
Supplement: S1 Table — Elevated cholesterol is defined as TC ≥ 5 mmol/L; Elevated LDL-C is LDL-C ≥3 mmol/L; Low HDL is HDL-C <1.0 mmol/L in men and <1.3 mmol/L in women; elevated TGs is TGs ≥1.7 mmol/L and elevated non-HDL-C is non-HDL-C >3.4 mmol/L; results are presented as odds ratios with corresponding 95% confidence intervals; the models are adjusted for age, educational status, household socioeconomic status, smoking, alcohol intake, physical inactivity, fruits and vegetable intake and use of lipid lowering medication; *The BMI model had only WTHR it them will BMI was included in WC and WTHR model. (DOCX) [file pone.0316527.s002.docx]

S1 Table. Regional differences in the association of the various adiposity phenotypes with abnormal lipid fractions in the combined AWI-Gen cohort

| **Adiposity phenotype** | **Population group** | **Elevated total cholesterol** | **Elevated LDL-C** | **Low HDL-C** | **Elevated TGs** | **Elevated**  **non-HDL-C** |
| --- | --- | --- | --- | --- | --- | --- |
|  |  |  |  |  |  |  |
| BMI in kg/m^2^ | *West Africa* | 1.11 (1.05, 1.89) | 1.61 (1.13, 2.30) | 1.68 (1.45, 2.05) | 1.29 (1.08, 2.06) | 1.47 (1.09, 1.97) |
|  | *East Africa* | 1.21 (1.18, 1.69) | 2.13 (1.08, 4.21) | 1.73 (1.39, 2.37) | 1.92 (1.05, 3.51) | 1.53 (1.49, 2.28) |
|  | *South Africa* | 1.77 (1.15, 3.69) | 2.73 (1.46, 5.11) | 1.84 (1.22, 2.04) | 1.99 (1.51, 2.91) | 2.49 (1.39, 4.48) |
|  |  |  |  |  |  |  |
| WC in cm | *West Africa* | 1.34 (1.27, 2.35) | 1.64 (1.45, 1.93) | 1.09 (1.05, 1.15) | 1.42 (1.09, 2.55) | 1.23 (1.12. 2.22) |
|  | *East Africa* | 1.39 (1.19, 1.95) | 1.78 (1.42, 2.46) | 1.21 (1.10, 1.42) | 1.53 (1.19, 2.54) | 1.40 (1.29, 2.18) |
|  | *South Africa* | 1.52 (1.29, 3.04) | 1.91 (1.45, 1.97) | 1.25 (1.07, 1.55) | 2.12 (1.06, 4.25) | 1.47 (1.09, 2.01) |
|  |  |  |  |  |  |  |
| WTHR | *West Africa* | 1.27 (1.09, 1.75) | 1.58 (1.22, 2.06) | 1.57 (1.42, 1.78) | 1.08 (1.06, 1.82) | 1.49 (1.13, 1.98) |
|  | *East Africa* | 1.43 (1.12, 1.82) | 1.88 (1.28, 2.76) | 1.63 (1.45, 1.86) | 2.49 (1.76, 3.53) | 1.69 (1.35, 2.12) |
|  | *South Africa* | 2.22 (1.37, 3.61) | 1.93 (1.17, 3.17) | 1.77 (1.47, 2.27) | 3.07 (2.01, 4.69) | 1.82 (1.22, 2.74) |

Elevated cholesterol is defined as TC ≥ 5 mmol/L; Elevated LDL-C is LDL-C ≥3 mmol/L; Low HDL is HDL-C <1.0 mmol/L in men and <1.3 mmol/L in women; elevated TGs is TGs ≥1.7 mmol/L and elevated non-HDL-C is non-HDL-C >3.4 mmol/L; results are presented as odds ratios with corresponding 95% confidence intervals; the models are adjusted for age, educational status, household socioeconomic status, smoking, alcohol intake, physical inactivity, fruits and vegetable intake and use of lipid lowering medication; *The BMI model had only WTHR it them will BMI was included in WC and WTHR model.
