# Supplementary material for: Obesity phenotypes and dyslipidemia in adults from four African countries: An H3Africa AWI-Gen study
Source: PLoS One. 2025 Jan 30;20(1):e0316527. doi: 10.1371/journal.pone.0316527 (PMC11781721; doi:10.1371/journal.pone.0316527)
Supplement: S2 Table — Data presented as prevalence ratios with West Africa as the reference group. West Africa includes Nanoro, Burkina Faso and Navrongo, Ghana sites; East Africa includes Nairobi, Kenya; and South Africa, Agincourt, Dikgale and Soweto sites; LDL-C, low density lipoprotein cholesterol; HDL-C, high density lipoprotein cholesterol and non-HDL-C, non-high-density lipoprotein cholesterol. (DOCX) [file pone.0316527.s003.docx]

S2 Table: Prevalence ratio of abnormal lipid levels sub-regional blocks with West Africa as the reference

|  | **Elevated**  **Total cholesterol** | **Elevated LDL-C** | **Low HDL-C** | **Elevated**  **Triglycerides** | **Elevated**  **Non-HDL-C** |
| --- | --- | --- | --- | --- | --- |
| **Women** |  |  |  |  |  |
| West Africa | Ref (1.00) | Ref (1.00) | Ref (1.00) | Ref (1.00) | Ref (1.00) |
| East Africa | 1.35 (1.30-1.39) | 1.45 (1.32-1.59) | 1.12 (0.98-1.27) | 1.56 (1.35-1.79) | 1.35 (1.24-1.47) |
| South Africa | 1.40 (1.35-1.45) | 1.61 (1.47-1.76) | 1.19 (1.05-1.35) | 1.58 (1.36-1.84) | 1.43 (1.31-1.45) |
| **Men** |  |  |  |  |  |
| West Africa | Ref (1.00) | Ref (1.00) | Ref (1.00) | Ref (1.00) | Ref (1.00) |
| East Africa | 1.16 (1.12-1.19) | 1.25 (1.12-1.39) | 1.04 (0.90-1.20) | 1.51 (1.31-1.74) | 1.19 (1.09-1.33) |
| South Africa | 1.23 (1.18-1.28) | 1.43 (1.31-1.57) | 1.14 (1.02-1.28) | 1.60 (1.35-1.89) | 1.25 (1.15-1.35) |
| **Combined sample** |  |  |  |  |  |
| West Africa | Ref (1.00) | Ref (1.00) | Ref (1.00) | Ref (1.00) | Ref (1.00) |
| East Africa | 2.98 (2.57, 3.46) | 1.18 (1.03, 1.35) | 1.87 (1.34, 2.43) | 2.49 (2.03, 3.08) | 2.64 (2.34, 2.99) |
| South Africa | 3.34 (2.86, 3.91) | 1.88 (2.64. 2.15) | 2.32 (1.87, 2.56) | 2.65 (2.19, 3.22) | 3.04 (2.68, 3.46) |

Data presented as prevalence ratios with West Africa as the reference group. West Africa includes Nanoro, Burkina Faso and Navrongo, Ghana sites; East Africa includes Nairobi, Kenya; and South Africa, Agincourt, Dikgale and Soweto sites; LDL-C, low density lipoprotein cholesterol; HDL-C, high density lipoprotein cholesterol and non-HDL-C, non-high-density lipoprotein cholesterol.
